# A Neutrophil Extracellular Traps Signature Predicts the Clinical Outcomes and Immunotherapy Response in Head and Neck Squamous Cell Carcinoma

**DOI:** 10.3389/fmolb.2022.833771

**Published:** 2022-02-18

**Authors:** Naifei Chen, Dongsheng He, Jiuwei Cui

**Affiliations:** ^1^ Cancer Center, The First Hospital of Jilin University, Changchun, China; ^2^ Department of Medical Oncology, The First Hospital of Putian, Teaching Hospital, Fujian Medical University, Putian, China

**Keywords:** HNSCC, nets, prognosis, immune microenvironment, immunophenoscore, anticancer drugs

## Abstract

**Background:** Neutrophil extracellular traps (NETs) play an important role in the occurrence, metastasis and immune escape of cancers. This study aimed to investigate NET-related genes, their clinical prognostic value and their correlation with immunotherapy and anticancer drugs in patients with head and neck squamous cell carcinoma (HNSCC).

**Methods:** Differentially expressed NET-related genes in HNSCC were identified based on multiple public databases. To improve the clinical practicability and avoid overfitting, univariable, least absolute shrinkage and selection operator (LASSO) and multivariable Cox algorithms were used to construct a prognostic risk model. A nomogram was further used to explore the clinical value of the model. Internal and external validation were conducted to test the model. Furthermore, the immune microenvironment, immunophenoscore (IPS) and sensitivity to anticancer drugs in HNSCC patients with different prognostic risks were explored.

**Results:** Six NET-related genes were screened to construct the risk model. In the training cohort, Kaplan–Meier (K-M) analysis showed that the overall survival (OS) of low-risk HNSCC patients was significantly better than that of high-risk HNSCC patients (*p* < 0.001). The nomogram also showed a promising prognostic value with a better C-index (0.726 vs 0.640) and area under the curve (AUC) (0.743 vs 0.706 at 3 years, 0.743 vs 0.645 at 5 years) than those in previous studies. Calibration plots and decision curve analysis (DCA) also showed the satisfactory predictive capacity of the nomogram. Internal and external validation further strengthened the credibility of the clinical prognostic model. The level of tumor mutational burden (TMB) in the high-risk group was significantly higher than that in the low-risk group (*p* = 0.017), and the TMB was positively correlated with the risk score (R = 0.11; *p* = 0.019). Moreover, the difference in immune infiltration was significant in HNSCC patients with different risks (*p* < 0.05). Furthermore, the IPS analysis indicated that anti-PD-1 (*p* < 0.001), anti-CTLA4 (*p* < 0.001) or combining immunotherapies (*p* < 0.001) were more beneficial for low-risk HNSCC patients. The response to anticancer drugs was also closely correlated with the expression of NET-related genes (*p* < 0.001).

**Conclusion:** This study identified a novel prognostic model that might be beneficial to develop personalized treatment for HNSCC patients.

## Introduction

Head and neck cancer, the sixth most common malignancies worldwide, leads to unacceptable mortality, with more than 450,000 deaths reported in 2020 (International Agency for Research on Cancer and World Health Organization, 2021). As the most common primary component of head and neck cancer, the main risk factors for head and neck squamous cell carcinoma (HNSCC) are smoking, chronic exposure to alcohol, different forms of chewing tobacco (such as betel palm) and HPV infection (Gillison et al., 2015). Currently, the overall survival (OS) of HNSCC patients is not satisfactory even in the context of surgery, radiotherapy, chemotherapy, targeted therapy and immunotherapy. Increasing amounts of evidence highlights that distant failure is 20–30% in locoregionally advanced HNSCC patients, while the percentage of locoregional failure is approximately 40–50%. HNSCC has become a serious global public health problem (Samra et al., 2018; Muzaffar et al., 2021). Therefore, exploring novel therapeutic targets and developing a novel prognostic model to improve personalized treatment is urgent.

Neutrophils are the most abundant component of circulating immune cells and they play an irreplaceable role in the response against pathogens. The immune cells can release neutrophil extracellular traps (NETs) under *in vitro* stimulation or pathological conditions (Kolaczkowska and Kubes, 2013; Li et al., 2020; Németh et al., 2020). NETs are extracellular web-like structures consisting of mitochondrial and nuclear DNA fibers decorated with histones and granular antimicrobial enzymes, which have been recently considered a host defense mechanism to entrap, constrain and kill invasive bacteria and other pathogens (Pruchniak and Demkow, 2019). The process of classical NETs formation is termed “neutrophil extracellular trap-osis (NETosis)”, which has been defined as a unique form of regulated cell death distinguished from other programmed cell deaths, such as necroptosis, autophagy and apoptosis (Manda-Handzlik et al., 2020). NETs play a crucial role in the pathogenesis of various diseases, such as COVID-19, cystic fibrosis, small vessel vasculitis and cancers (Demkow, 2021).

Although NETs might theoretically exert antitumorigenic effects by trapping and killing cancer cells, increasing amounts of evidence indicates that NETs might exert pro-tumorigenic effects. NETs can be triggered by cancer cells and cancer-associated fibroblasts, which directly or indirectly facilitate the proliferation and metastasis of cancer cells by hijacking the antimicrobial immune system (Park et al., 2016). It has been suggested that NETs are related to the recurrence of abdominal cancers and a poor prognosis of patients with colorectal cancer and Ewing sarcoma (Berger-Achituv et al., 2013; Richardson et al., 2017; Kanamaru et al., 2018). Moreover, NETs have also been reported to be enriched in the liver metastases of patients with colon and breast cancers, and serum NETs might predict the occurrence of liver metastases in patients with early-stage breast cancer (Yang et al., 2020).

Epithelial mesenchymal transition (EMT) arms cancer cells with motility and invasiveness, and NETs can induce this transition. The web-like structure might increase the adhesive ability of cancer cells and awaken dormant cancer cells (Ireland and Oliver, 2020; Teijeira et al., 2020; Yang and Liu, 2021). Furthermore, NETs enhance the immune escape of cancer cells. It has been suggested that the NETs might to reduce the curative effect of immune checkpoint inhibitors (ICIs) and chimeric antigen receptor T-cell (CAR-T) immunotherapy (Teijeira et al., 2020; Volkov et al., 2021). In summary, NETs have become a new field of investigation in oncology. Nevertheless, NET-related genes, their prognostic value and their relationship with immunotherapy in HNSCC remain largely unknown.

In the present study, we first constructed a prognostic risk model related to NETs in HNSCC based on multiple public databases, and internal and external validations were used to assess the accuracy of the risk model. Furthermore, the clinical value, immune microenvironment and drug sensitivity based on the model were investigated. To the best of our knowledge, there are no previous studies exploring NET-related genes, their prognostic value or their relationship with the immune microenvironment in HNSCC. This study found that NET-related genes might be potential prognostic markers and therapeutic targets in HNSCC patients and could be used to further improve the efficacy of treatment in patients with HNSCC through personalized treatment.

## Materials and Methods

### Data Acquisition

The mRNA sequencing data (FPKM) of HNSCC patients (44 head and neck normal samples and 502 HNSCC samples) in The Cancer Genome Atlas (TCGA) database and mRNA sequencing data (FPKM) of 55 head and neck normal samples (salivary gland) in the Genotype-Tissue Expression (GTEx) database were obtained from UCSC Xena (https://gtexportal.org/home/). The clinical data of the HNSCC patients were downloaded from the TCGA database. A total of 170 NET-related genes were obtained from previous studies and are shown in Supplementary Table S1 (Dwyer et al., 2014; Papayannopoulos, 2018).

### Data Processing

The sequencing data in TCGA and GETx were transformed with log2 (FPKM+1) and normalized with the limma R package, and 99 normal head and neck samples and 502 HNSCC samples were integrated (Ritchie et al., 2015). The 170 NET-related genes were matched with the mRNA sequencing data, and the limma R package was used to screen out the differentially expressed NETs genes (DEGs) with log2 (fold change) > 1 and adjusted *p* < 0.05 (Wu et al., 2020).

### Construction and Assessment of the Prognostic Risk Score Model

The expression of NET-related genes in HNSCC patients were integrated with the corresponding survival data. The patients with HNSCC in the TCGA database were randomly separated into training and testing groups at a ratio of 8:2. In the training group (*n* = 400), the NET-related genes were included in a univariate Cox regression analysis (*p* < 0.1) to identify candidate genes (Jiao et al., 2021). Then, differentially expressed candidate genes -related NETs were identified by intersecting the DEGs and candidate genes. The least absolute shrinkage and selection operator (LASSO) Cox regression algorithm was applied to avoid overfitting. Furthermore, multivariable Cox regression analysis was used to compute the coefficient of the prognostic risk score model. The HNSCC patients in the training group were divided into high- and low-risk cohorts based on the median risk score. The Kaplan-Meier method, receiver operating characteristic (ROC) curve, distribution of risk score and survival status were used to evaluate the efficiency of the prognostic risk model. Moreover, gene ontology (GO) and kyoto encyclopedia of genes and genomes (KEGG) analysis of the screened genes was performed by employing the “clusterProfiler” R package (Liu et al., 2021).

### Internal and External Validation of the Multigene Prognostic Model

To further evaluate the value of the prognostic risk model of HNSCC patients, a testing cohort (*n* = 99) and a whole cohort (*n* = 499) in the TCGA database were used to conduct internal validation. The mRNA sequencing and clinical data of HNSCC patients (*n* = 108) from the E-MTAB-8588 dataset in the ArrayExpress database were used to conduct an external validation, and the “sva” R package was used to diminish the batch effect of different datasets (Leek et al., 2012). Internal and external validation was conducted based on the medium risk score in the training cohort.

### Construction and Assessment of the Nomogram for Patients With Head and Neck Squamous Cell Carcinoma

To further weight the possibility of the risk score based on genes related to NETs being an independent prognostic parameter, the risk score of HNSCC patients was integrated with the corresponding clinical parameters (age, sex, grade, stage, margin status, chemotherapy, and radiotherapy) in the training cohort. The parameters were included in a univariate Cox regression algorithm to screen out the characteristics correlated with overall survival (OS) in HNSCC patients with a *p* value less than 0.05. A multivariable Cox regression algorithm was further used to identify independent prognostic parameters. Then, a nomogram was constructed based on the parameters. To weigh the capability of the clinical prognostic model to forecast an individual’s OS, the concordance index (C-index), ROC curve, calibration plot and decision curve analysis (DCA) were calculated. The nomogram was further validated for testing the overall and external cohorts for internal and external validation.

### Relationship Between Immune Infiltration and Prognostic Risk Score in Patients With Head and Neck Squamous Cell Carcinoma

The single nucleotide variant (SNV) data in the TCGA database were downloaded to calculate the tumor mutational burden (TMB) for each HNSCC patient. Spearman’s algorithm was used to analyze the correlation between the risk score and TMB. Furthermore, the “Cell Type Identification by Estimating Relative Subsets of RNA Transcripts (CIBERSORT)” deconvolution algorithm with 1,000 permutations was applied to quantify 22 types of tumor-infiltrating lymphocytes (TILs) in the microenvironment of low- and high-risk patients with HNSCC with a *p* value less than 0.05 (Becht et al., 2016). Moreover, the data of the immunophenoscore (IPS) of the HNSCC patients were downloaded from The Cancer Immunome Atlas (TCIA, https://tcia.at/), and the immunotherapy response for anti-PD-1 and anti-CTLA4 in low- and high-risk patients with HNSCC were further investigated (Charoentong et al., 2017).

### Exploration of Drug Sensitivity Based on the Prognostic Model

To explore the anticancer drugs targeted to the NET-related genes that constructed the prognostic model, the sensitivity data of anticancer drugs approved by the United States Food and Drug Administration were downloaded from the CellMiner database (https://discover.nci.nih.gov/cellminer/) (Foy et al., 2017). Pearson analysis was used to explore the correlation between anticancer drug sensitivity and NET-related genes to construct the prognostic risk model (Reinhold et al., 2019).

## Results

### Identification of Differentially Expressed Genes Related to Neutrophil Extracellular Traps in Head and Neck Squamous Cell Carcinoma

**FIGURE 1:**
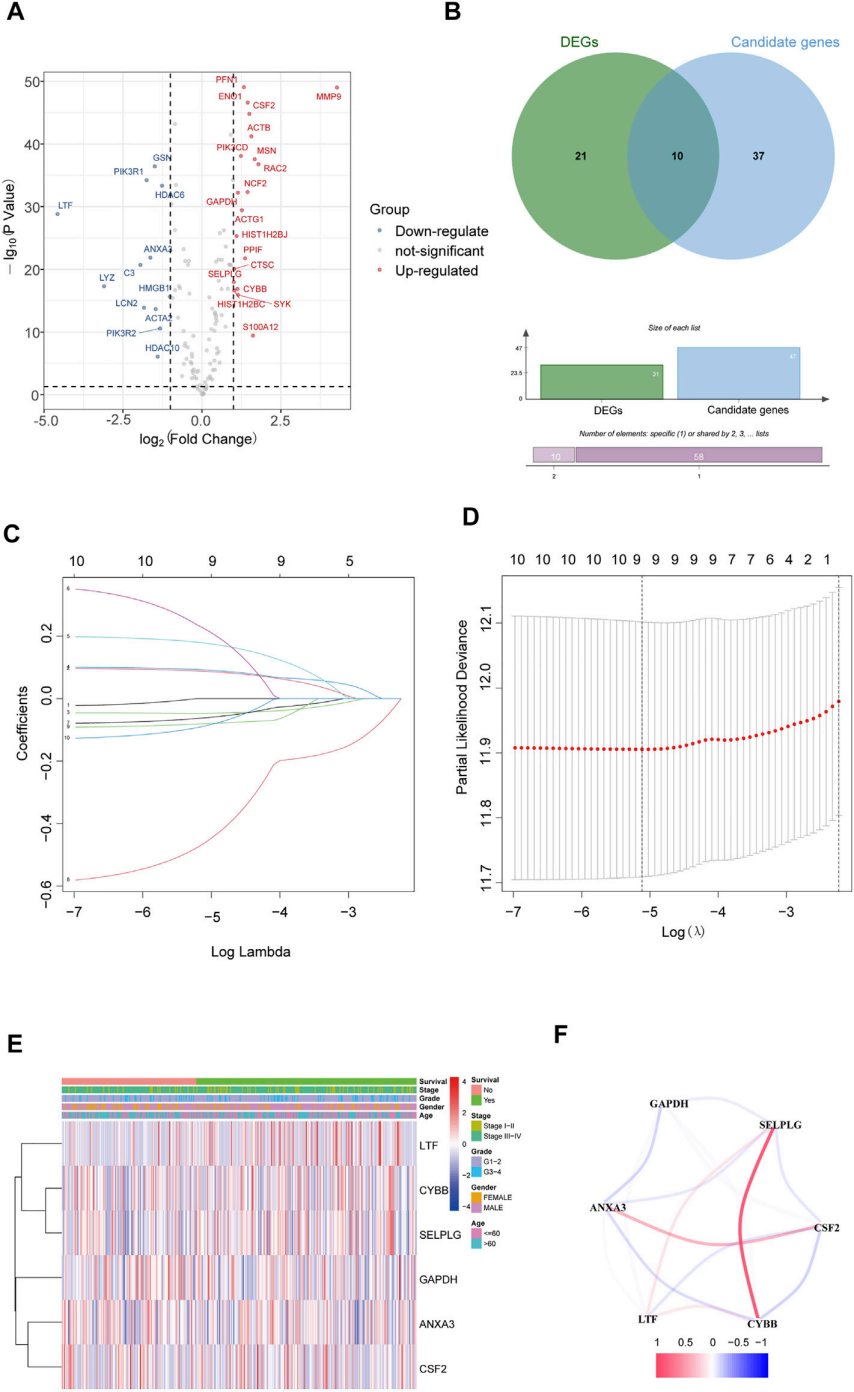
Identification of NET-related signatures in HNSC**C. (A)** Differentially expressed genes related NETs in HNSCC. **(B)** Differentially expressed candidate genes related NETs in HNSCC. **(C)** LASSO coefficient profiles of 9 genes related NETs. **(D)** Cross-validation for tuning parameter selection in the proportional hazards model. **(E)** The heatmap of the 6 genes related NETs. **(F)** The correlation of the 6 genes related NETs.

The gene expression of NETs in HNSCC was filtered by matching mRNA sequencing data of HNSCC and 170 NETs. Then, a total of 31 differentially expressed genes (19 upregulated and 12 downregulated) that were related to NETs were screened out in HNSCC by using the limma R package (Figure 1A).

### Construction and Assessment of a Prognostic Model Based on Gene-Related Neutrophil Extracellular Traps in Head and Neck Squamous Cell Carcinoma

**FIGURE 2:**
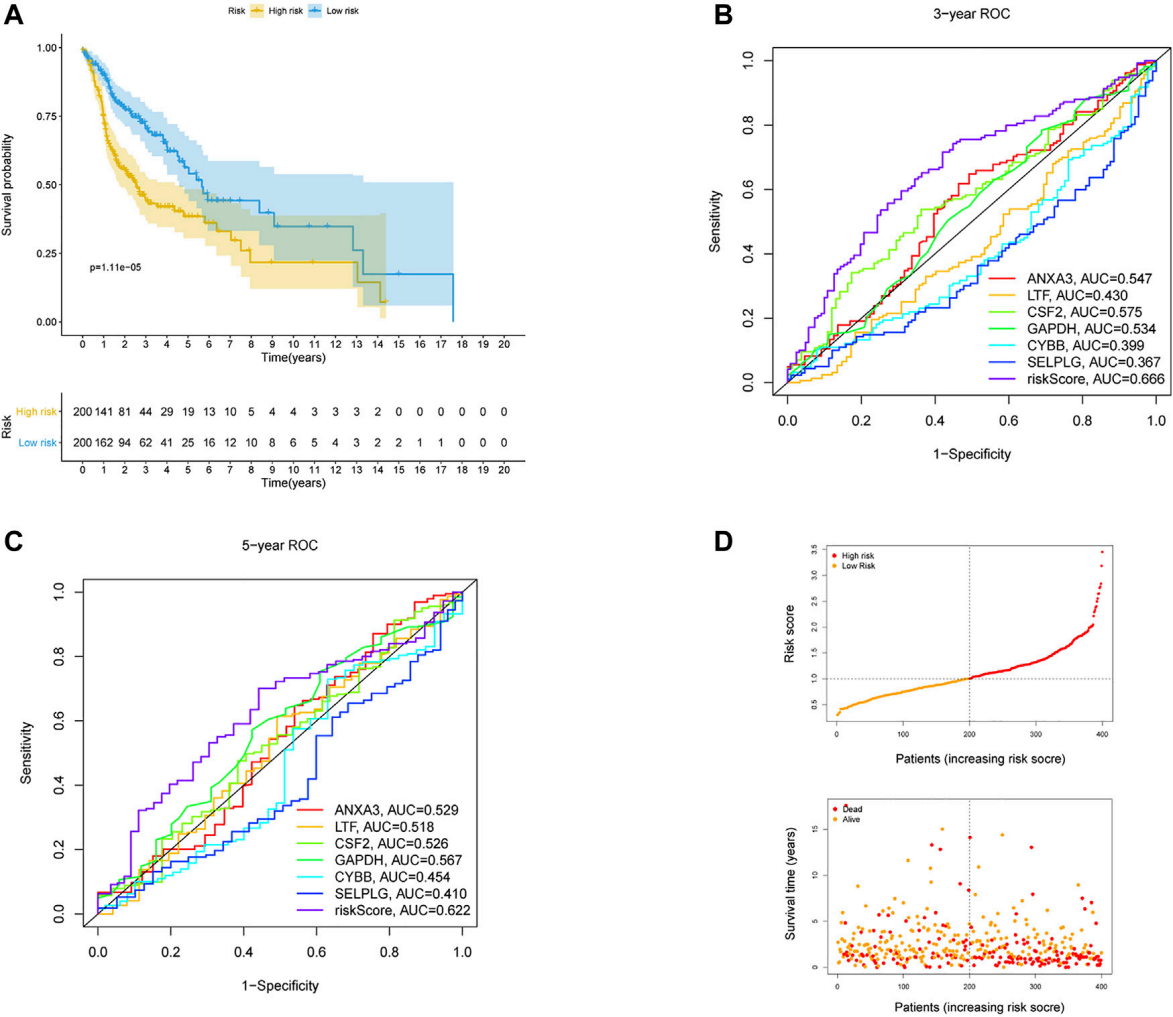
Validation of the risk prognostic model in training cohort. **(A)** K-M survival analysis of the model. **(B,C)** ROC curves analysis of the model at 3 and 5 years. **(D)** Distribution of risk score and survival status based on the prognostic model.

The 170 NET-related genes was matched with the expression and clinical data of HNSCC patients. To construct a convincing prognostic risk model, HNSCC patients were randomly separated into training (n = 400) and testing cohorts (n = 99) at a ratio of 8:2. In the training cohort, a univariate Cox regression algorithm was used to investigate the candidate genes related to NETs and 10 differentially expressed candidate genes were filtered (Figure 1B). The 10 genes were included in the LASSO Cox regression algorithm to avoid overfitting (Figure 1C), and cross validation was conducted, which filtered out 9 prognostic genes (Figure 1D). To further improve the clinical practicability, 6 gene signatures were purified by multivariable Cox regression analysis and used to build a prognostic risk model (Figure 1E). The risk score was calculated as follows: Risk score= (0.116 × ANXA3 expression level) + (-0.062 × LTF expression level) + (0.120 × CSF2 expression level) + (0.184 × GAPDH expression level) + (CYBB × 0.306 expression level) + (-0.604 × SELPLG expression level). The correlations of the 6 genes are shown in Figure 1F. Moreover, the credibility of the model was assessed. In the K-M analysis, the OS between patients with high and low risk was significantly different (*p* < 0.001) (Figure 2A). The area under the curve (AUC) values for the ROC curves at 3 and 5 years were 0.666 and 0.622, respectively (Figures 2B,C). Furthermore, the gene function and potential pathways of the 6 genes are displayed in Supplementary Figure S1. The most highly enriched function of the 6 screened genes were neutrophil degranulation and neutrophil activation involved in immune response. KEGG pathway analysis suggested that the 6 genes might participate in NETs formation or HIF−1 signaling pathway. The HIF-1 has been reported to regulate neutrophil from anti-tumor N1 to pro-tumor N2, the latter might promote the NETs formation (Guglietta et al., 2016; Triner and Shah, 2019).

### Internal and External Validation of the Prognostic Risk Model

**FIGURE 3:**
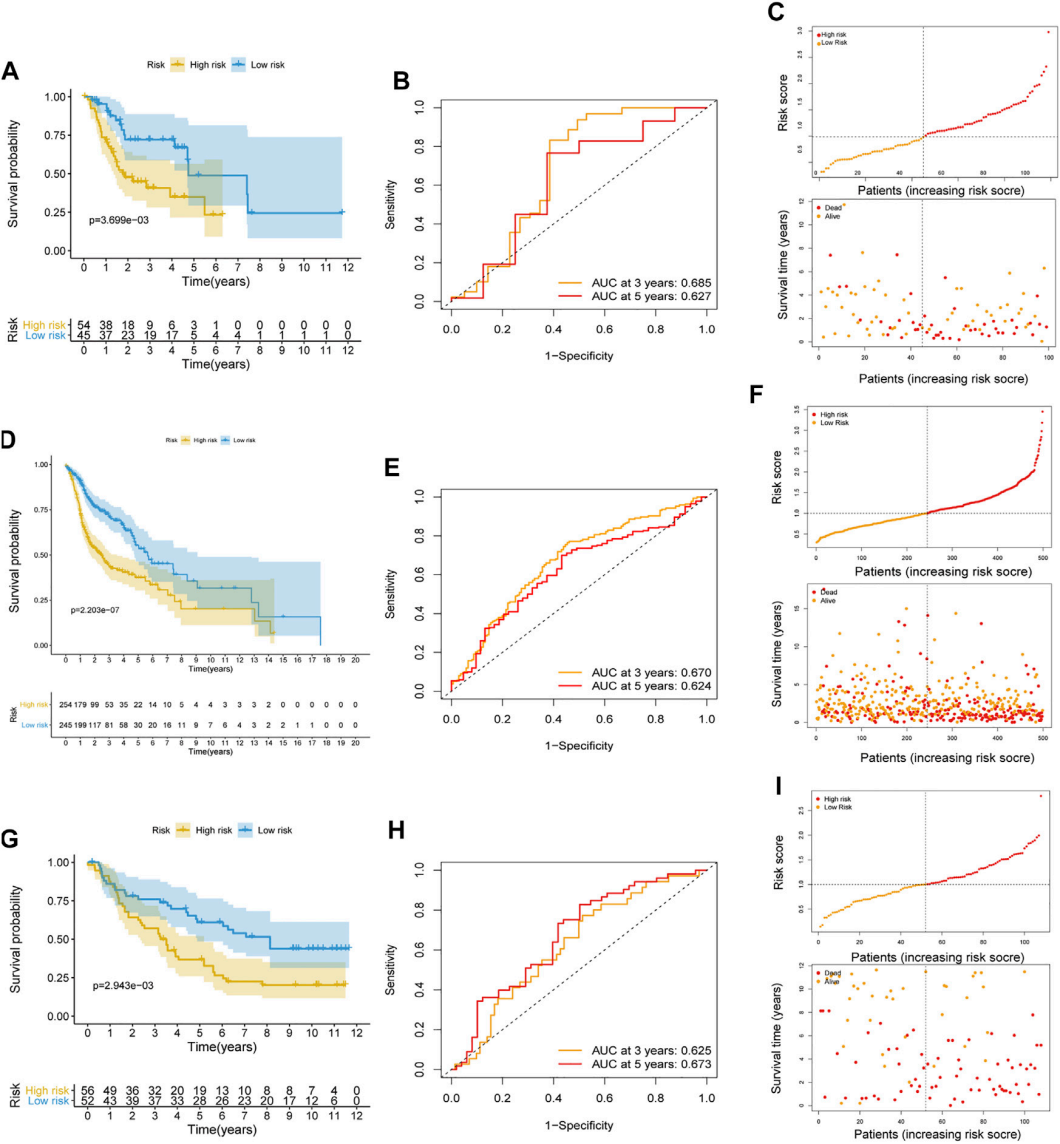
Internal and external validation of the risk prognostic model. **(A–C)** K-M analysis, ROC curves, distribution of risk score and survival status in testing cohort. **(D–F)** K-M analysis, ROC curves, distribution of risk score and survival status in entire cohort. **(G–I)** K-M analysis, ROC curves, distribution of risk score and survival status in external validated cohort.

To further weight the predictive capability of the model in the training cohort, the testing cohort and entire cohort were used for internal validation, and the E-MTAB-8588 dataset was used for external validation. The HNSCC patients were separated into high- (n = 54) and low- (n = 45) risk groups in the testing cohort based on the medium risk score in the training cohort. The difference in OS between the two groups was significant (*p* < 0.01) (Figure 3A), and the AUC values at 3 and 5 years were 0.685 and 0.627, respectively (Figures 3B,C). In the entire cohort, the difference in OS between the high-risk group (n = 254) and the low-risk group (n = 245) was significant (*p* < 0.001) (Figure 3D). In addition, the AUCs at 3 and 5 years were 0.670 and 0.624, respectively (Figures 3E,F). Similar to the internal validation, external validation also showed acceptable K-M analysis (*p* < 0.01) (Figure 3G) and AUCs (0.625 at 3 years; 0.673 at 5 years) that increased year by year (Figures 3H,I).

### Construction and Assessment of a Clinical Prognostic Model

**FIGURE 4:**
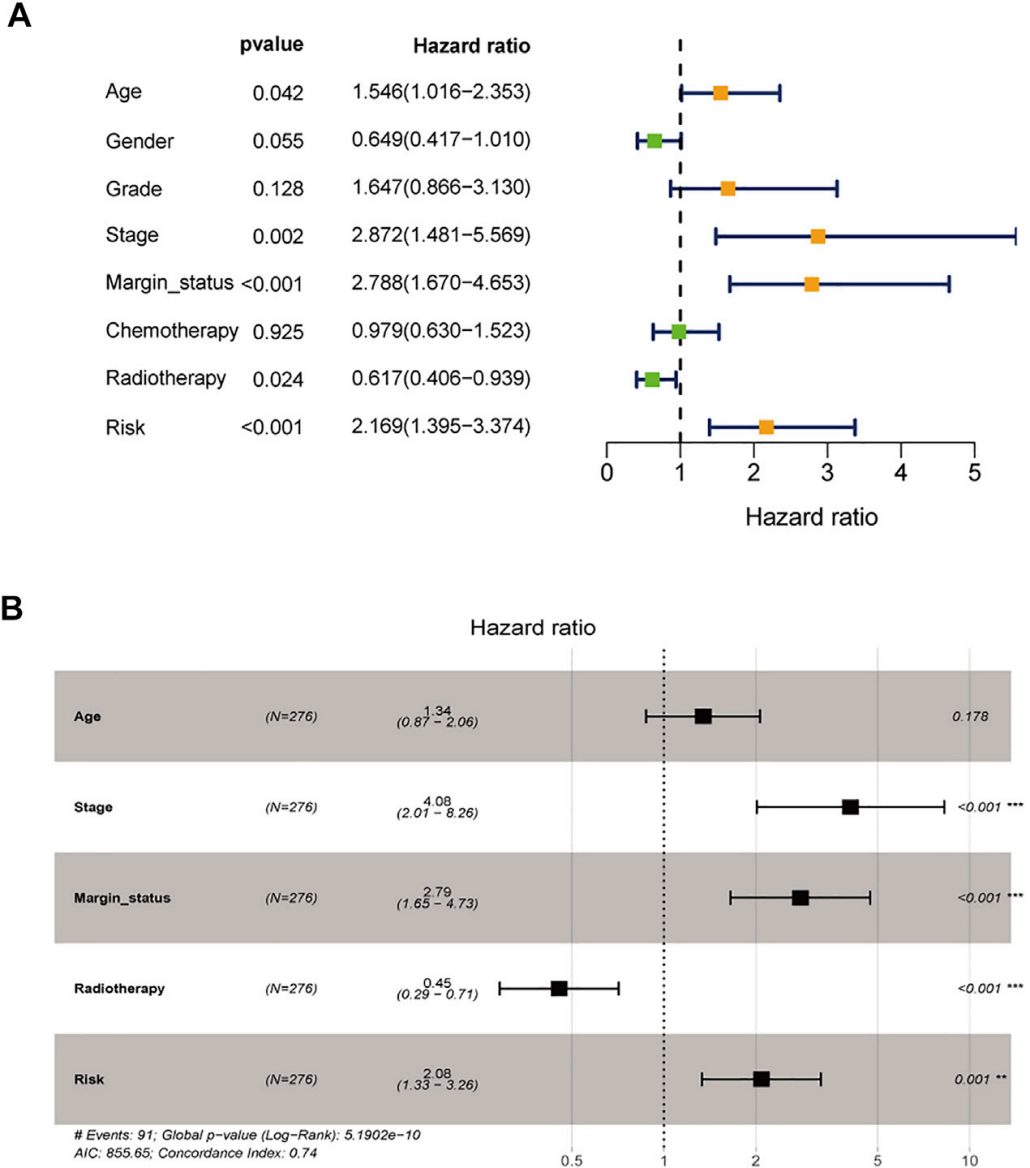
Identification of independent prognostic parameters in HNSCC. **(A)** The univariate Cox regression analysis of clinical parameters. **(B)** The multivariate Cox regression analysis of clinical parameters.

**FIGURE 5:**
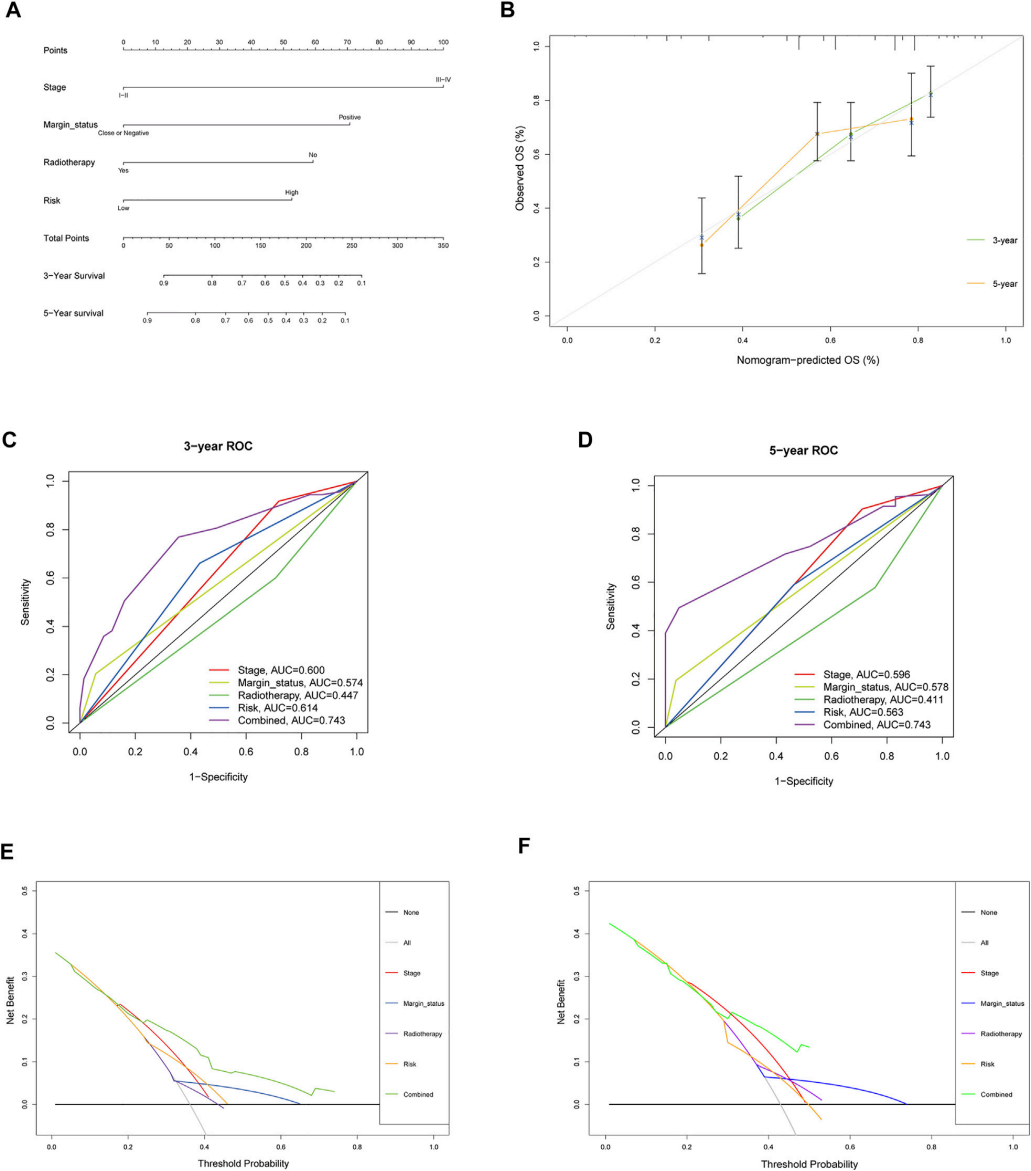
Nomogram to predict 3- and 5- year OS and its validation in training cohort. **(A)** Nomogram to predict 3- and 5- year OS of HNSCC patients. **(B)** Calibration plot analysis to assess the accuracy of nomogram to predict 3- and 5- year OS in HNSCC patients. **(C-D)** ROC curves to assess the accuracy of nomogram to predict 3- and 5- year OS in HNSCC patients. **(E,F)** DCA to assess the accuracy of nomogram to predict 3- and 5- year OS in HNSCC patients.

To assess the possibility of the risk score being an independent prognostic factor, the risk score of HNSCC patients in the training cohort was matched with the clinical parameters (age, sex, grade, stage, margin status, radiotherapy and risk score), and 276 individuals were included in the univariate Cox regression analysis. Age, stage, margin status, radiotherapy and risk score were correlated with the prognosis of the HNSCC patients (Figure 4A). A multivariable Cox algorithm was used to further screen out four independent prognostic parameters of the HNSCC patients (stage, margin status, radiotherapy and risk score) with a *p* value less than 0.001 (Figure 4B). Based on the four parameters, a nomogram with an optimistic C-index (0.726) was built to predict an individual’s prognosis at 3 and 5 years (Figure 5A). ROC curve analysis showed satisfactory AUC values at 3 and 5 years (0.743 and 0.743, respectively) (Figures 5B,C). The actual curve was also close to the ideal curve in the calibration plot at 3 and 5 years (Figures 5D,E). Moreover, DCA also further confirmed the creditability of the prognostic accuracy of the nomogram (Figures 5F,G).

### Internal and External Validation of the Clinical Prognostic Model

**FIGURE 6:**
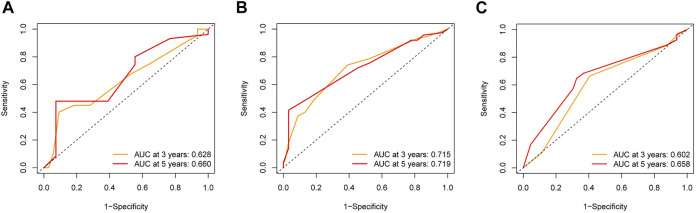
The validation of nomogram in internal and external cohorts. **(A)** ROC curves to assess the accuracy of nomogram to predict 3- and 5- year OS in testing cohort. **(B)** ROC curves to assess the accuracy of nomogram to predict 3- and 5- year OS in entire cohort. **(C)** ROC curves to assess the accuracy of nomogram to predict 3- and 5- year OS in external cohort.

To further explore the credibility of the nomogram in the training cohort, the risk score in the testing, entire cohort and E-MTAB-8588 dataset were matched with the corresponding clinical parameters for internal and external validation. In the testing cohort, the C-index was 0.667, and the AUC values at 3 and 5 years were 0.628 and 0.660, respectively, increasing year by year (Figure 6A). In the entire cohort, the C-index was 0.686, and the AUC values at 3 and 5 years were 0.715 and 0.719, respectively, increasing year by year (Figure 6B). In the externally validated cohort, the C-index was 0.590 when the margin status data were missing, and the AUC values at 3 and 5 years were 0.602 and 0.658, respectively, increasing year by year (Figure 6C). The internal and external validations together suggested the reliability of the nomogram in the training cohort. Moreover, the risk score is significantly correlated with T stage, perineural invasion, extracapsular spread and different cancer statue and subtype (*p* < 0.05) (Supplementary Figure S2).

### Relationship Between Immune Infiltration and Prognostic Risk Score in Patients With Head and Neck Squamous Cell Carcinoma

**FIGURE 7:**
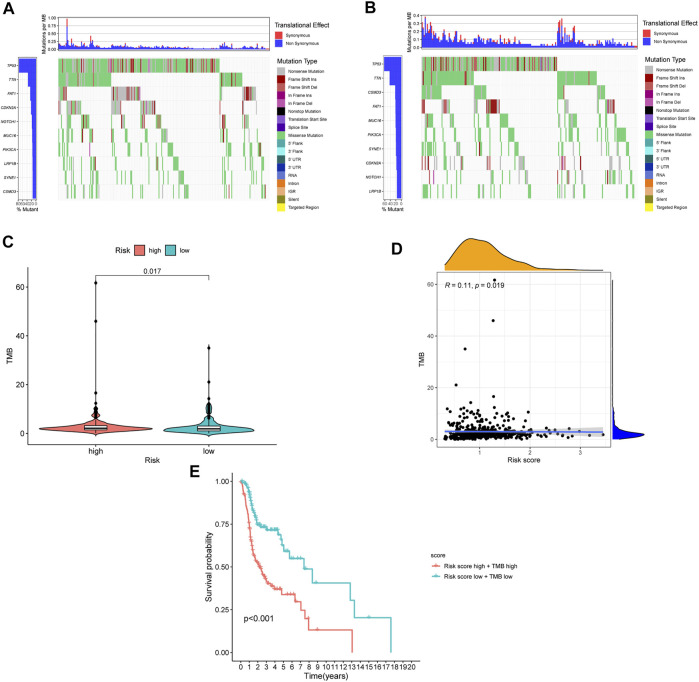
The differences of TMB in high and low risk HNSCC patients. **(A)** The TMB in high risk HNSCC patients. **(B)** The TMB in low risk HNSCC patients. **(C)** The difference of TMB was significant in HNSCC patients with different risk. **(D)** The TMB was positively correlated with the risk score in patients with HNSCC. **(E)** The OS of the HNSCC patients of high risk and high TMB were lower than those in HNSCC patients with low risk and low TMB.

**FIGURE 8:**
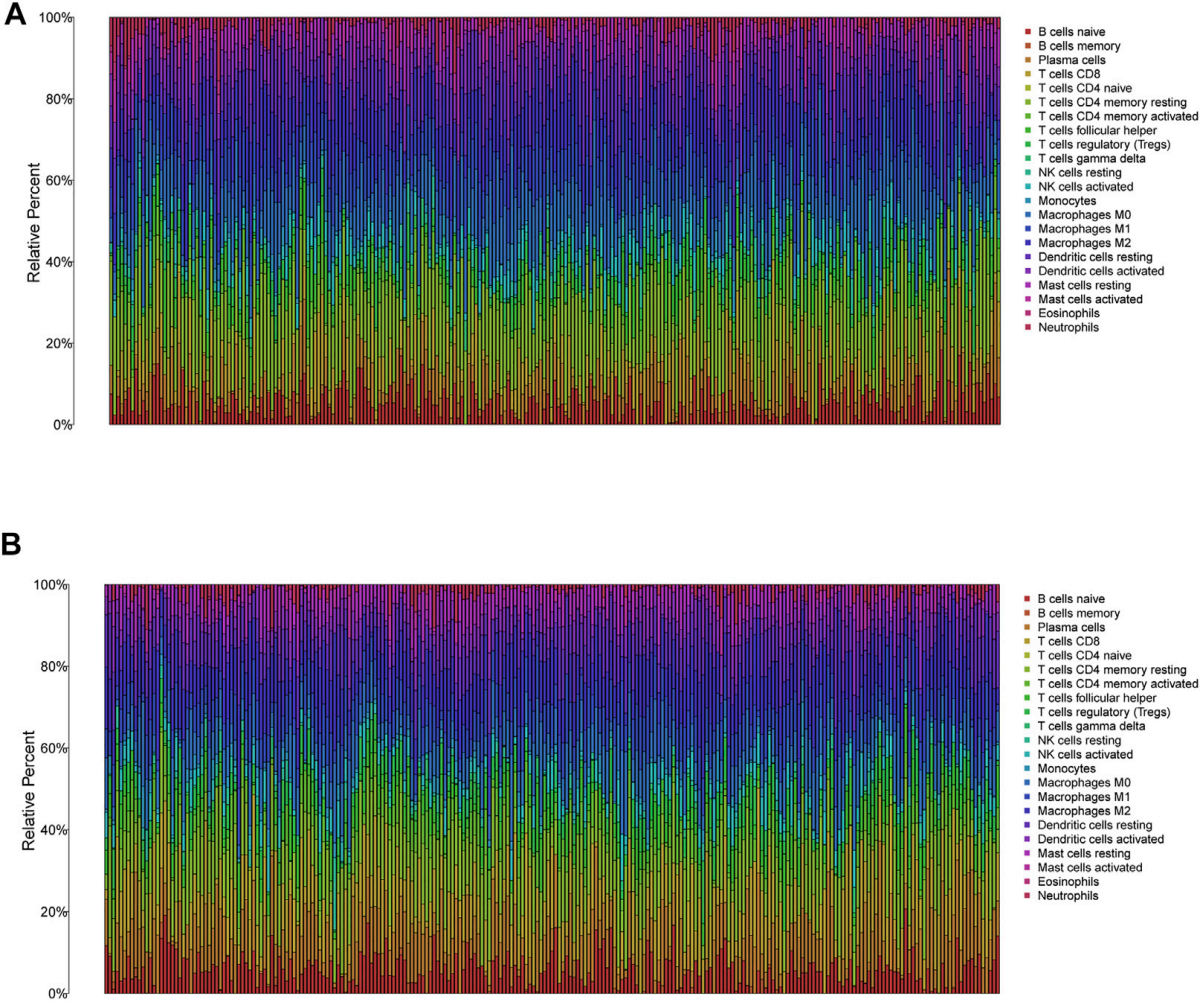
The immune infiltration of 22 immune cell types in high and low risk patients with HNSCC. **(A)** The immune infiltration of 22 immune cell types in high risk patients with HNSCC. **(B)** The immune infiltration of 22 immune cell types in low risk patients with HNSCC.

The TMB has been indicated to be a market for the curative effect of immune checkpoint inhibitors. The TMB of the high- and low-risk groups of HNSC patients is shown in Figures 7A,B. The TMB difference was significant between the two groups (*p* = 0.017) (Figure 7C), and the risk score was positively correlated with TMB (R = 0.11; *p* = 0.019) (Figure 7D). Moreover, the OS of the high-risk and high-TMB group was lower than that of the low-risk and low-TMB group, with a *p* value less than 0.001 (Figure 7E). However, a TMB with a high level might fail to accurately predict the immune checkpoint blockade response across all cancer types, and the immune microenvironment in HNSCC patients was further explored based on the risk predictive model related to NETs (Figure 8).

**FIGURE 9:**
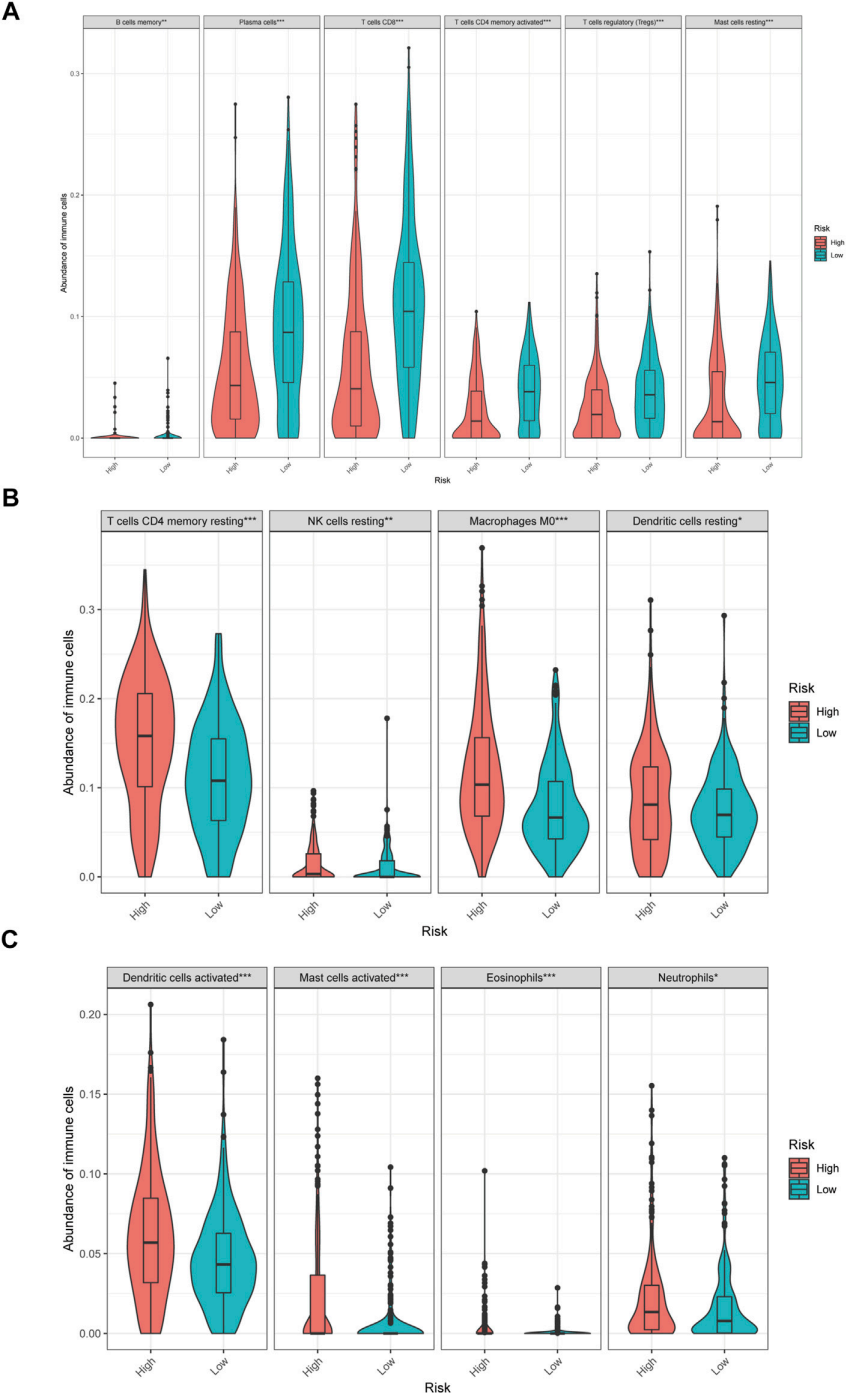
The different immune infiltration in the high and low risk HNSCC patients. **(A)** The expression of six types of immune cells is higher in low risk group compared with high risk group. **(B,C)** The expression of eight types of immune cells is higher in high risk group compared with low risk patients.

In low-risk patients with HNSCC, higher expression of markers of memory B cells (*p* < 0.01), plasma cells (*p* < 0.001), CD8 T cells (*p* < 0.001), CD4 memory activated T cells (*p* < 0.001), regulatory T cells (Tregs) (*p* < 0.001), and resting mast cells (*p* < 0.001) was observed (Figures 11A–E), while higher expression of markers of CD4 memory resting T cells (*p* < 0.001), resting NK cells (*p* < 0.01), M0 macrophages (*p* < 0.001), resting dendritic cells (*p* < 0.05), activated dendritic cells (*p* < 0.05), activated mast cells (*p* < 0.001), eosinophils (*p* < 0.001), and neutrophils (*p* < 0.05) were observed than in the high-risk group (Figure 9).

**FIGURE 10:**
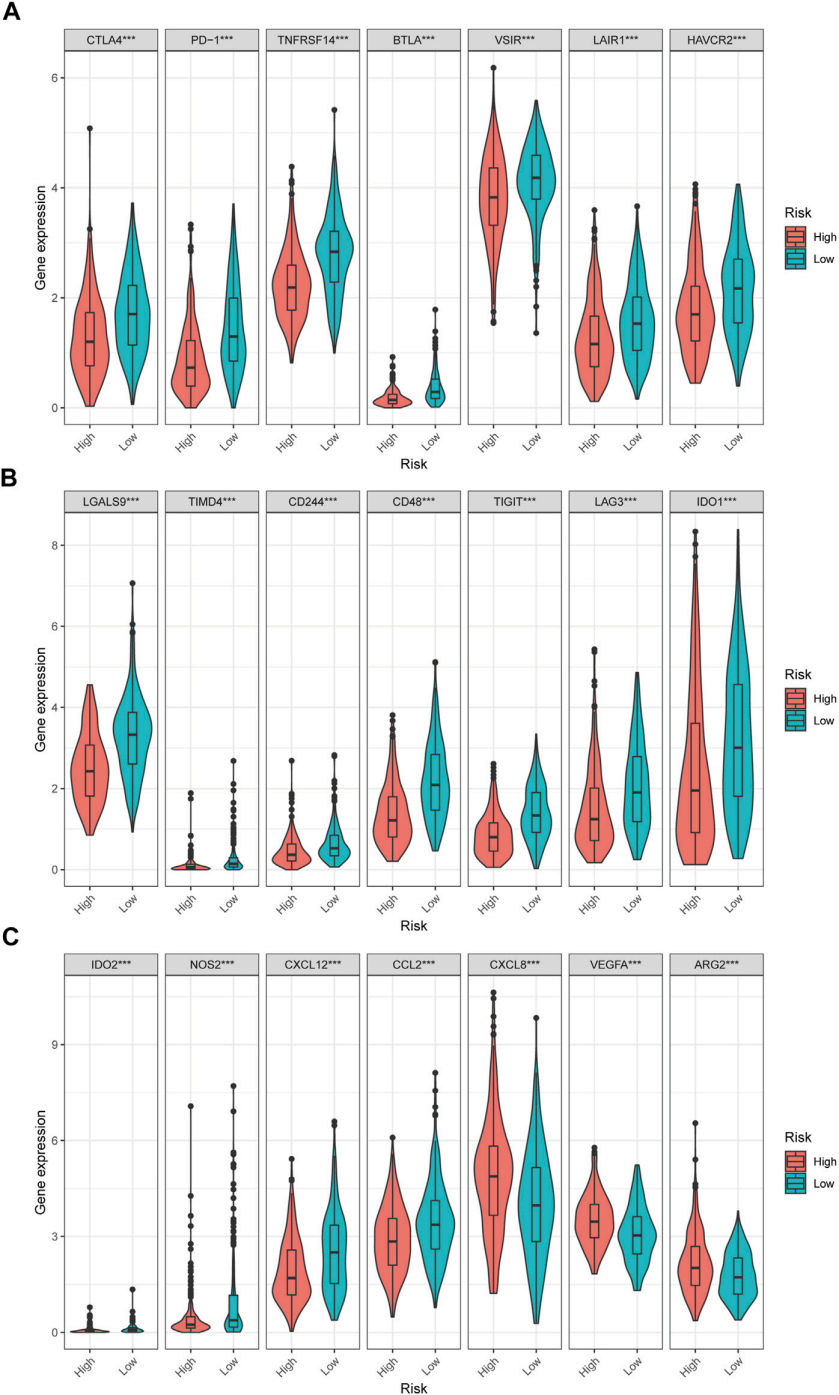
The difference of immune checkpoint genes in the high and low risk HNSCC patients.

**FIGURE 11:**
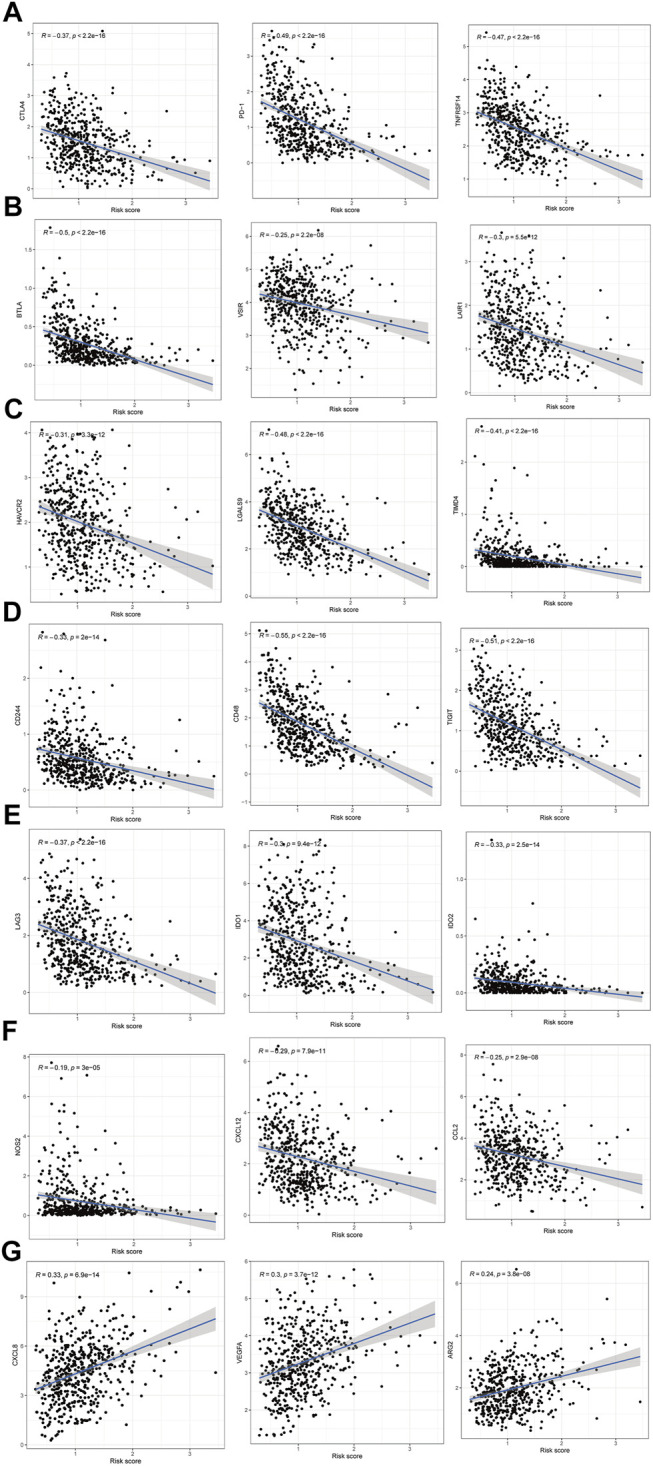
The correlations between the immune checkpoint genes and risk score in HNSCC patients. **(A–F)** The immune checkpoint genes was negatively correlated with the risk score. **(G)** The immune checkpoint genes was positively correlated with the risk score.

Moreover, differences in immune checkpoint genes in low- and high-risk HNSCC patients were investigated. The expression levels of CTLA4, PD-1, TNFRSF14, BTLA, VSIR, LAIR1, HAVCR2, LGALS9, TIMD4, CD244, CD48, TIGIT, LAG3, IDO1, IDO2, NOS2, CXCL12 and CCL2 were higher in the low-risk group than in the high-risk group (*p* < 0.001). In contrast, the high-risk group had higher CXCL8, VEGFA, and ARG2 expression than the low-risk group (*p* < 0.001) (Figure 10). The corresponding correlations between the immune checkpoint genes and risk scores are shown in Figure 11.

### Immunophenoscore Analysis of Head and Neck Squamous Cell Carcinoma in the High and Low Risk Groups

**FIGURE 12:**
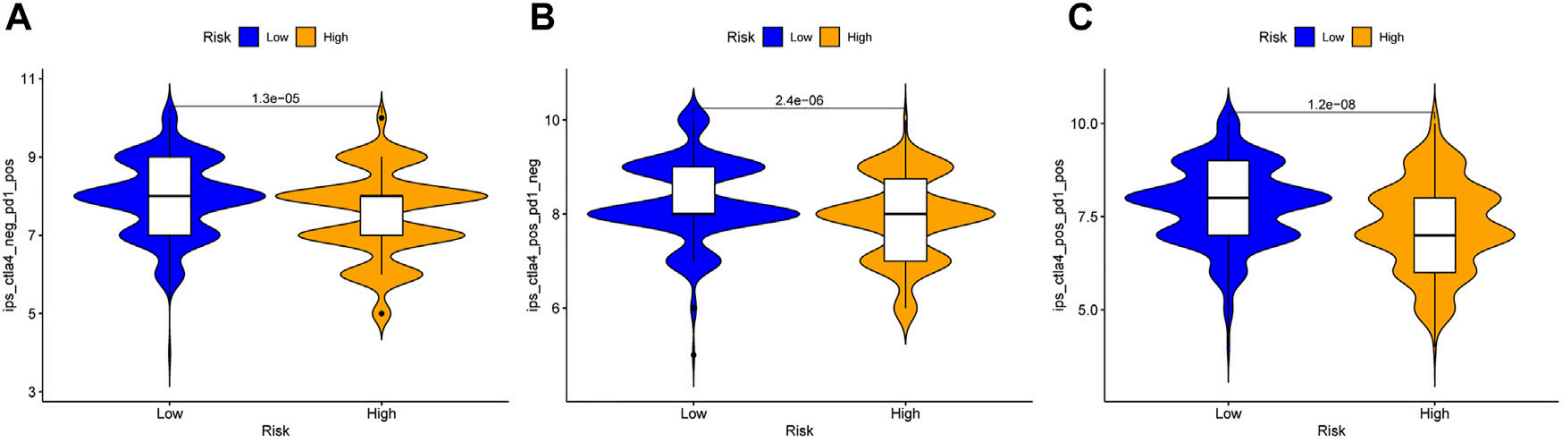
Immunophenoscore analysis in the high and low risk HNSCC patients. **(A)** Low-risk group might receive more benefits from anti-PD-1 therapy than the high-risk HNSCC patients. **(B)** Low-risk group might receive more benefits from anti-CTLA4 therapy than the high-risk HNSCC patients. **(C)** Low-risk group might receive more benefits from combined immunotherapy than the high-risk HNSCC patients.

To further strengthen the credibility of the immune checkpoint genes based on the risk predictive model, the IPS were analyzed in different risk groups. The IPS might be useful to predict the curative effect of immunotherapy. As shown in Figures 12A–C, the three subgroups shown the IPS is significantly higher in low risk group than that in high risk group, which indicated that the low-risk group might receive more benefits from anti-PD-1 therapy, anti-CTLA4 therapy or combined immunotherapy with anti-PD-1 and anti-CTLA4 than the high-risk HNSCC patients.

### Exploration of Drug Sensitivity Based on the Prognostic Model

**FIGURE 13:**
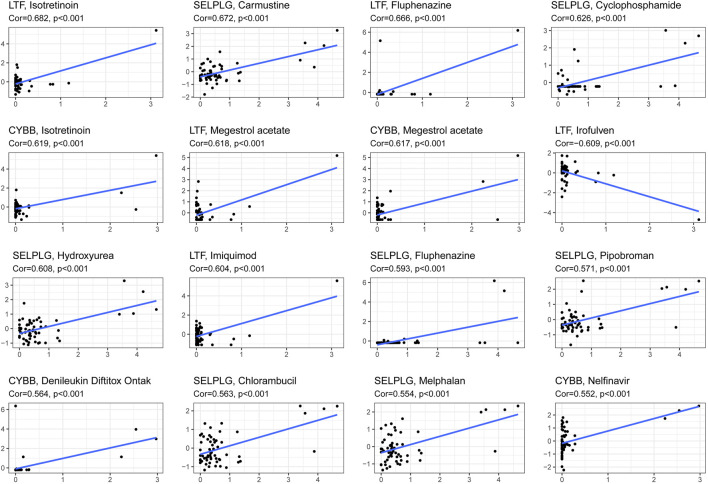
The correlation between genes related to NETs to construct the prognostic model and drug sensitivity.

This model can be used not only to predict the survival of HNSCC patients but also to differentiate the difference in the immune microenvironment in HNSCC patients with different levels of risk. Therefore, it is necessary to investigate anticancer drugs targeting the model. The 16 representative correlation analyses are shown in Figure 13. LTF, SELPLG and CYBB were most strongly correlated with anticancer drugs. In the representative correlation analyses, LTF and CTBB were both positively correlated with isotretine, and SELPLG was positively correlated with carmustine.

## Discussion

Convincing evidence has shown that NETs participate in the initiation and development of cancers. NETs are formed when neutrophils develop NETosis, and the web-like structure has been found to play a procancer role in cancer invasion, evasion and metastasis (Demkow, 2021). The OS of HNSCC patients is unsatisfactory even in the context of comprehensive treatment, and the 5-years survival rate of HNSCC patients is further reduced by recurrence, at a 50% rate for the locally advanced stage (Muzaffar et al., 2021). To the best of our knowledge, NETs, an emergent hotspot in oncology, have not been comprehensively investigated in previous studies related to HNSCC.

In the study, a risk prognostic model related to NETs in HNSCC was constructed, the optimistic clinical value of the model was shown by multiple validations. Moreover, the tumor immune microenvironment, immune checkpoint genes, IPS and drug sensitivity in HNSCC based on the prognostic model were further analyzed, which all indicated the potential value of the model related to NETs in immunotherapy of HNSCC patients.

In the present study, we first systematically explored the correlation between NET-related genes and HNSCC. To improve its credibility, the sequencing data in the TCGA and GTEx databases were integrated, which maximally diminished the imbalance between cancer samples and noncancer samples. The 6 NET-related genes were screened out by univariate, LASSO, and multivariable logistic regression algorithms to construct the prognostic risk model, which avoided overfitting while strengthening the clinical practicability of the model. The majority of the 6 signature genes in the risk model have been found to be involved in multiple cancers. However, the role of some of these genes in HNSCC is still unclear.

Annexin A3 (ANXA3), an important member of the Annexin multigene family, plays a pivotal role in signaling pathways and malignant biological behaviors of cancer cells, such as proliferation and apoptosis. The abnormal expression of ANXA3 is correlated with the development, occurrence, metastasis and drug resistance of cancers (Park et al., 2005; Harashima et al., 2008; Bandorowicz-Pikuła et al., 2012). The gene expression was exclusively restricted to neutrophils, it’s reported that the ANXA3 participate in the formation of NETs in several diseases such as rheumatoid arthritis and systemic lupus erythematosus (Chapman et al., 2019; Toufiq et al., 2020). Lactotransferrin (LTF) is an important member of the transferrin gene family, and it encodes a glycoprotein that is a major iron-binding protein and is widely expressed in saliva, trachea, milk, nasal secretions and neutrophil particles (Bournazou et al., 2010). Some studies have shown that LTF has an antitumor function and can inhibit the proliferation and metastasis of cancer cells, and the expression of this gene is downregulated in multiple types of cancers (Lee et al., 2003; Ni et al., 2020). The LTF has been indicated to suppress the formation and release of NETs, which might be correlated to the anticancer role of the gene (Okubo et al., 2016). Colony-stimulating factor 2 (CSF2) is a granulocyte macrophage-colony stimulating factor that can stimulate the production of monocytes and granulocytes. This gene is associated with neutrophil counts and has been indicated to be an oncogene in several cancers, such as colon cancer and urothelial carcinoma (Lee et al., 2016; Xu et al., 2019). The colony-stimulating factors increase the number of neutrophils and induce their activation, NETs can be released by the systemic release of colony-stimulating factors in cancers, which might be one of carcinogenic effects of CSF2 (Demers and Wagner, 2013). Glyceraldehyde-3-phosphate dehydrogenase (GAPDH) is a key glycolytic pathway-related enzyme that catalyzes redox reactions. However, convincing evidence suggests that GAPDH may play a nonenzymatic role, which is correlated with DNA repair, autophagy and apoptosis (Hara and Snyder, 2006; Colell et al., 2007; Azam et al., 2008). It has been reported that GAPDH is overexpressed in cancer cells, promoting their proliferation and metastasis (Liu et al., 2017). The GAPDH has been indicated to participate in the formation of NETs, the production of NETs increase when the expression level of GAPDH is up-regulated (Dwyer et al., 2014; Agraz-Cibrian et al., 2019). Cytochrome b-245 beta chain (CYBB, also known as NOX2) is a compound enzyme complex that is only expressed in myeloid cells such as macrophages and neutrophilic granulocytes (Martner et al., 2019). The generation of NETs, an early effect of NOX2 activation in neutrophils, is linked to NOX2 activation (Singel and Segal, 2016). Selectin P ligand (SELPLG, also known as PSGL-1) is mainly expressed in inflammatory and immune cells and participates in the recruitment of inflammatory and immune cells to the site of inflammation by tethering and rolling (Laszik et al., 1996). SELPLG act a positive effect to enhances neutrophil recruitment and NET formation, its deficiency might affect neutrophil function and immune cell differentiation and therefore act on tumor growth (Coffelt et al., 2015; Yago et al., 2018).

The HNSCC patients have been divided high- and low risk-groups based on the 6 genes related to NETs, which suggested that the difference of NETs in different risk groups. The OS of HNSCC patients with low NETs was significantly better than that of HNSCC with high NETs, K-M and AUCs at 3 and 5 years showed acceptable values in the training cohort. Furthermore, the internal and external validation improve the credibility of the model. Overall, the practicability of the model in the training cohort was validated by using multiple methods.

To further investigate the clinical prognostic value of the model related to NETs, the risk score of HNSCC was integrated with clinical parameters. Univariate and multivariable Cox regression algorithms showed that the risk score could be an independent prognostic parameter of HNSCC patients, and the risk score has a synergistic effect with other clinical parameters to improve the value of the nomogram to calculate an individual prognosis. The nomogram used to predict individual prognosis in this study was more effective than that in previous studies with a superior C-index (0.726 vs 0.640) and AUC for 3 (0.743 vs 0.706) and 5 years (0.743 vs 0.645) (He et al., 2021). In the internal and external validation cohorts, the C-index and AUC for 3 and 5 years also showed satisfactory values, which further strengthened the credibility of the clinical prognostic model. The results further indicated that NETs might acts a synergistic effect with other clinical parameters to predict the clinical outcomes of HNSCC patients.

Moreover, NETs have been suggested to play an important role in immune evasion, wrapping and protecting cancer cells from the anticancer effect of neighboring immune cells such as NK cells and CD8^+^ T cells in tumor immune microenvironment (TME) (Ireland and Oliver, 2020; Teijeira et al., 2020). NETs acts a “physical barrier” to cover the cancer cells and reduce the curative effect of ICIs and CAR-T. It has been reported that the responsiveness of tumor to PD-1 plus CTLA-4 dual checkpoint blockade can be improved by inhibiting NETs. Moreover, the efficiency of CAR-T might be increased by reducing the NETs in TME (Teijeira et al., 2020; Volkov et al., 2021). Furthermore, the NETs has been suggested to be a biomarker to prognose the response of anti-PD-1 therapy in melanoma based on the significant effect of NETs in immunotherapy (Jensen et al., 2021). Therefore, further exploration of the correlation between the model based on NETs and the immune microenvironment of HNSCC patients is necessary.

TMB has been reported to be a marker for predicting the curative effect of immunotherapy in cancer patients (Chan et al., 2019). In this study, the TMB of HNSCC patients was positively correlated with the risk score, which indicated that the prediction result of TMB and NETs is inconsistent. It has been reported that a high TMB fails to predict the immune checkpoint blockade response across all cancers, which is consistent with our result (McGrail et al., 2021). Therefore, the immune microenvironment in high- and low-risk HNSCC patients needs to be further investigated.

In the present study, the differences in immune infiltration and immune checkpoint genes in high- and low-risk HNSCC patients were illustrated, we found the differences of immune infiltration and immune checkpoint genes were significant. Regulatory T cells (Tregs) are a specialized subpopulation of T cells that act to suppress immune response in some cancers such as ovarian carcinoma, prostate cancer, and non-small cell lung cancer (Curiel et al., 2004; Tao et al., 2012; Flammiger et al., 2013). However, the role of Tregs is associated with a favorable outcome in colorectal carcinoma, Triple-negative breast cancer, and HNSCC (Salama et al., 2009; Bron et al., 2013; Song et al., 2015). In the TME of HNSCC patients, the infiltration abundance of Tregs in high risk group is lower than that in low risk group, the favorable role of Tregs in HNSCC might be correlated with the anti-inflammatory capacity of Tregs which restric inflammation-related carcinogenesis (Shang et al., 2015). The difference of TME in HNSCC patients might be beneficial for improving personalized treatment and immunotherapy effects by using different immune checkpoint inhibitors. Furthermore, the difference of IPS in high- and low-risk HNSCC patients further strengthen the possibility.

Certainly, the present study had several potential limitations. The in-depth molecular mechanisms of the NET-related genes used to construct the prognostic model must be further verified in experimental studies. Moreover, this study was based only on research data from public databases, which might contribute to selection bias. Thus, a multicenter and large-scale study is necessary to further validate the clinical utility of our model.

## Conclusion

In summary, for the first time, this study identified a novel prognostic model of HNSCC patients based on 6 NET-related genes. Furthermore, the value of the model is promising for predicting the individual prognosis with other clinical parameters, immunotherapy, and drug sensitivity, which suggests that this novel model related to NETs might be beneficial in improving individualized treatment, thereby improving the curative effect for HNSCC patients.

## Data Availability

The datasets used and analyzed for this study were obtained from TCGA (https://portal.gdc.cancer.gov/), UCSC Xena (https://xena.ucsc.edu/), ArrayExpress (https://www.ebi.ac.uk/arrayexpress/), TCIA (https://tcia.at/) and CellMiner database (https://discover.nci.nih.gov/cellminer/).

## Supplementary Material

Supplementary Figure S1 Representative results of GO and KEGG analyses. **(A)** The GO analysis of the 6 screened genes. **(B)** The potential signaling pathway of the screened 6 genes.

Supplementary Figure S2 The correlation between risk score and different clinical characteristics in HNSCC patients.

Supplementary Table S1 The NET-related genes.
