# Supplementary figures and images for: A Neutrophil Extracellular Traps Signature Predicts the Clinical Outcomes and Immunotherapy Response in Head and Neck Squamous Cell Carcinoma
Source: Front Mol Biosci. 2022 Feb 18;9:833771. doi: 10.3389/fmolb.2022.833771 (PMC8894649; doi:10.3389/fmolb.2022.833771)

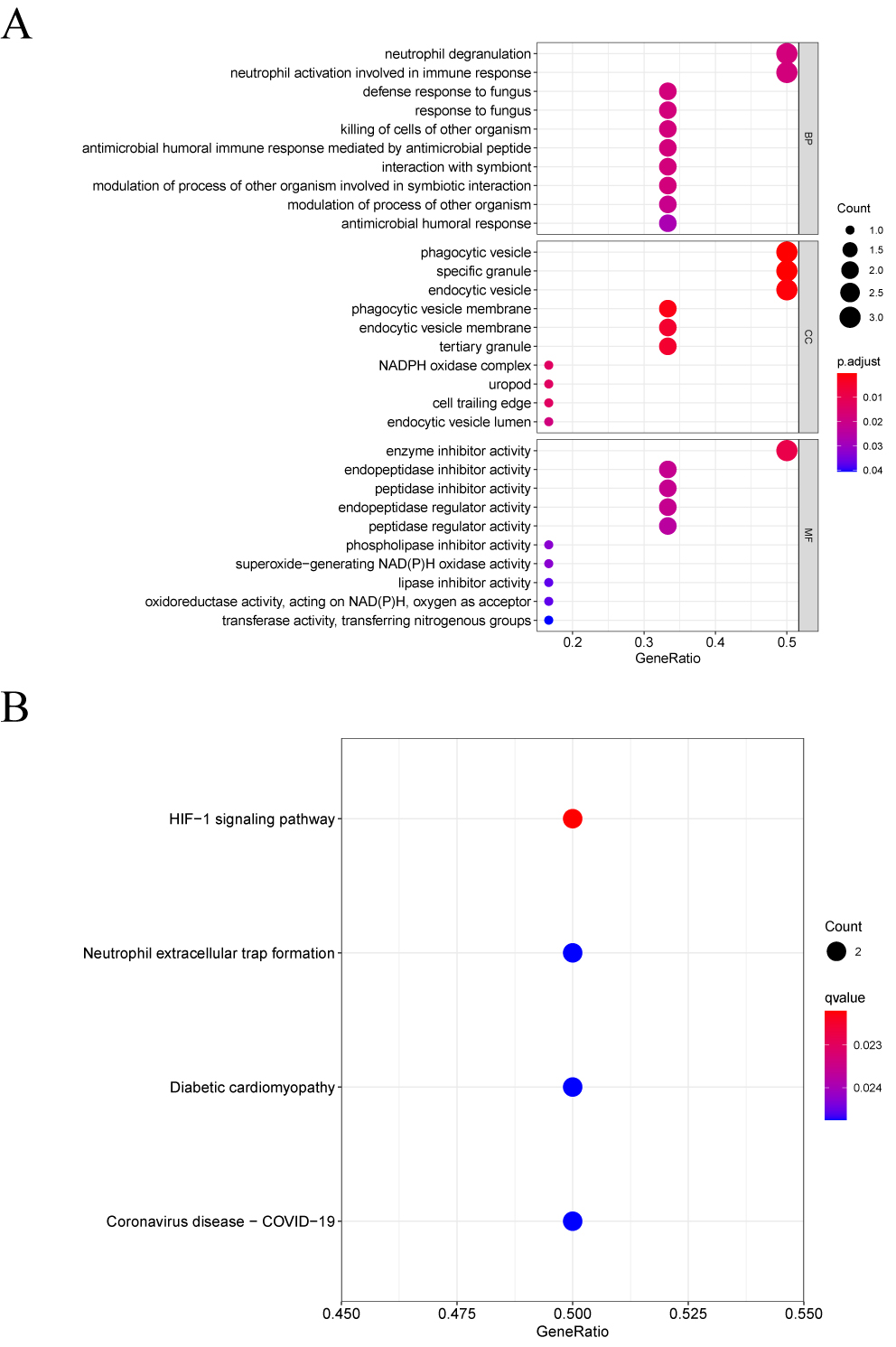

Supplement: Supplementary file 1 [file Image1.JPEG]

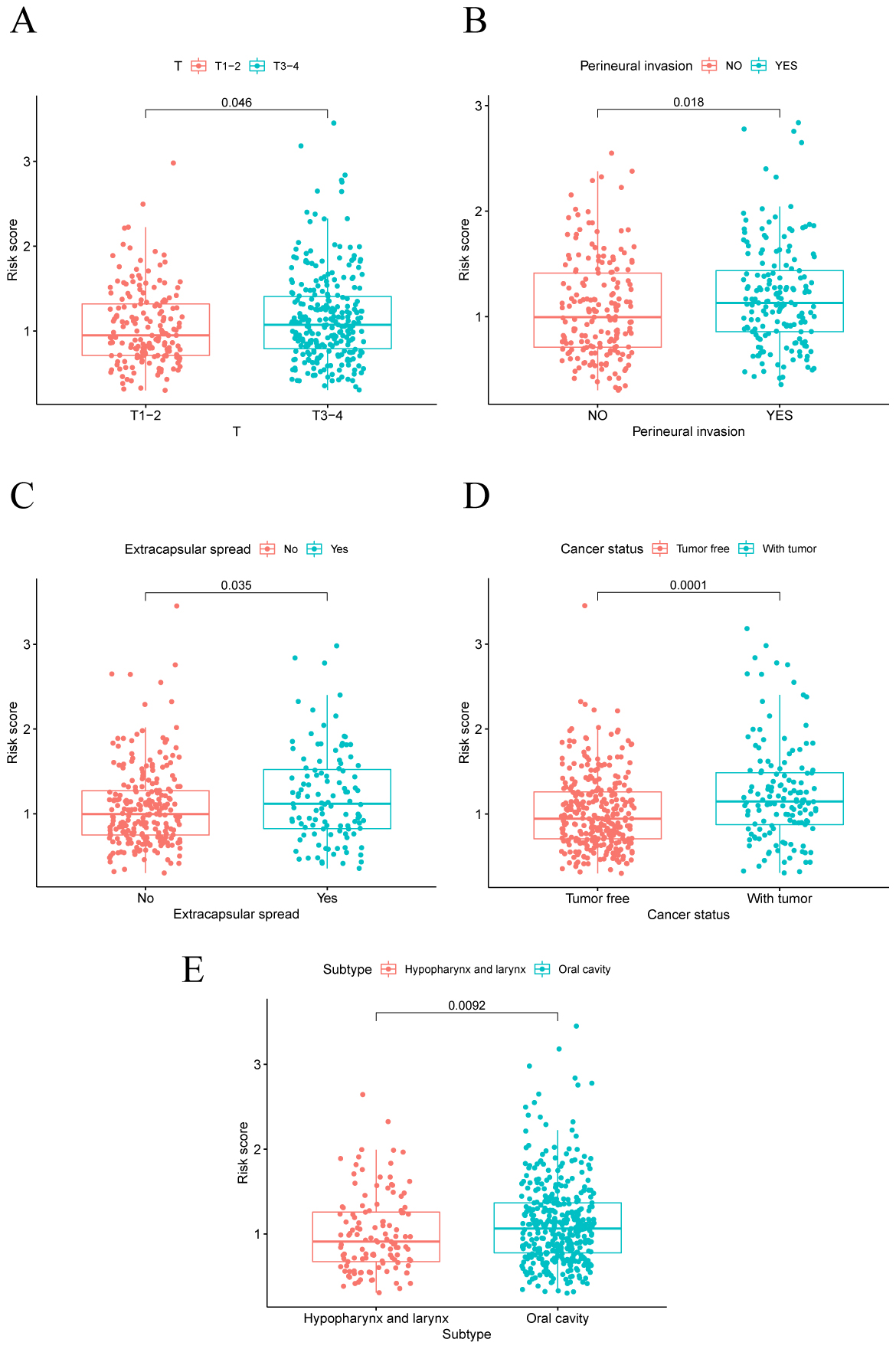

Supplement: Supplementary file 2 [file Image2.JPEG]
